# Membranous or Hypermobile Stapes Footplate: A New Anatomic Site Resulting in Third Window Syndrome

**DOI:** 10.3389/fneur.2020.00871

**Published:** 2020-08-20

**Authors:** Arun K. Gadre, Ingrid R. Edwards, Vicky M. Baker, Casey R. Roof

**Affiliations:** ^1^Department of Otolaryngology—Head and Neck Surgery, Geisinger Medical Center, Geisinger Commonwealth School of Medicine, Danville, PA, United States; ^2^Heuser Hearing Institute and Speech and Language Academy, Louisville, KY, United States; ^3^Clinical Audiologist, Department of Otolaryngology—Head and Neck Surgery and Communication Sciences, University of Louisville, Louisville, KY, United States

**Keywords:** head trauma, vertigo, stapes footplate defect, hypermobile stapes, third window syndrome, perilymph fistula, gray-scale invert function CT scan

## Abstract

**Objectives:** To describe a potentially underappreciated pathology for post-traumatic persistent intractable dizziness and third window syndrome as well as the methods to diagnose and surgically manage this disorder.

**Study Design:** Observational analytic case studies review at a tertiary care medical center.

**Methods:** Patients suffering persistent dizziness following head trauma and demonstrating Tullio phenomena or Hennebert signs are included. All had reportedly normal otic capsules on high resolution temporal bone CT scans (CT). The gray-scale invert function was used to visualize the stapes footplate, which helped determine the diagnosis. Gray-scale inversion can be used to improve visualization of temporal bone anatomy and pathologic changes when diagnoses are in doubt. A search to check for the presence of perilymph leakage was performed in all cases. This was accomplished using intraoperative Valsalva maneuvers. Fat grafting of round and oval windows was performed.

**Results:** Over an 11-year period between January 2009 and December 2019, 28 patients (33 ears) were treated. Follow-up with balance testing and audiograms were performed 6–8 weeks following surgery. Follow-up ranged from 6 months to 7 years. Prior to surgery all patients reported dizziness in response to loud sounds and/or barometric pressure changes. Seven out of 33 ears had demonstrable perilymph leakage into the middle ear; the rest (26 ears) appeared to have membranous or hypermobile stapes footplates. Membranous stapes footplates were better visualized using the invert function on CT. Thirteen patients had a fistula sign positive bilaterally while 15 had unilateral pathology. Twenty-four of the 28 patients (85.7%) showed both subjective and objective improvement following surgery. No patients suffered from a deterioration in hearing.

**Conclusions:** A previously underappreciated membranous or hypermobile stapes footplate can occur following head trauma and can cause intractable dizziness typical of third window syndrome (TWS). Durable long term success can be achieved by utilizing fat graft patching of the round and oval windows. High resolution temporal bone CT scans using the gray-scale inversion (invert) function can assist in preoperative diagnosis.

## Introduction

Acute dizziness may be associated with head trauma, which can even be trivial (1, 2). Following head trauma, persistent, intractable, and sometimes intermittent vertigo or dizziness constitutes a diagnostic and therapeutic challenge. The nature of the injury is often mild and may not result in concussion or loss of consciousness. Many of these patients seek treatment without relief, and when all else fails, they may be inappropriately labeled as suffering from post-concussive syndrome (PCS), or chronic traumatic encephalopathy (CTE). A presumptive diagnosis of perilymph fistula with leakage of perilymph may also be entertained.

We have identified a unique cohort of patients who develop immediate or delayed symptoms which overlap quite closely with those suffering from third window syndrome (TWS) of which superior semicircular canal dehiscence syndrome is best characterized (3). Symptoms include disabling dizziness or vertigo often induced by barometric pressure changes as well as loud sounds. Vertigo spells are generally persistent and are often associated with cognitive deficits. During exploratory surgery we found that presumed leakage of perilymph at the oval or round windows is relatively uncommon; however small or large defects covered over by a membrane are often observed in the stapes footplate. Alternatively, a hypermobile stapes footplate may be discovered.

Our article summarizes clinical and surgical findings in a series of 28 patients. Three illustrative cases have been included. Vestibular testing using bithermal caloric testing is typically non-diagnostic or may show diminished function. However, positive Tullio, and Hennebert tests (fistula test) are often the first clue to diagnosis. The findings on cervical vestibular evoked myogenic potentials (cVEMP) is highly variable and needs to be investigated further. The “invert” function on high resolution CT is suggested as an additional means to identify stapes footplate defects prior to surgery. In a majority of cases fat-grafting of the round and oval windows appears to be curative without resulting in hearing loss.

## Methods

This study represents our experience over an 11-year period (January 1, 2009 through December 31, 2019). The procedures followed were in accordance with the ethical standards of the responsible committee on human experimentation and with the Helsinki Declaration. The Heuser Hearing Institute Institutional Review Board approved these observational analytic case studies (IRB IORG0006526). The Institutional Review Board granted a consent waiver and also approved the use of age and gender as deidentified data. Preliminary findings on the first 9 patients were presented at the 7th International Symposium on Meniere's Disease and Inner Ear Disorders (Rome, Italy October 17–20, 2015) and the Combined Otolaryngology Spring Meeting (COSM Scottsdale, AZ, January 18–20, 2018) but were not published. These cases are included in this scientific paper.

Patients suffering from persistent or intermittent incapacitating vertigo or dizziness following head trauma and demonstrating symptoms suggestive of ear fullness, fluctuating hearing loss, sound intolerance, and hyperacusis are included. Patients sometimes complain of autophony. In addition, several patients reported that the horizon was tilted. They variously described their symptoms as an inability to tolerate loud sounds. They also variously described being dizzy, lightheaded or vertiginous. During waking hours several patients describe a continuous sense of being on a “teeter-totter.” Others described being in a “funhouse,” “wobbly,” “foggy,” or describe a “fuzzy- sensation” with an inability to concentrate, where even slight movements of the head would make them dizzy. They all describe the need to hold on to a stable support to avoid falling during standing and ambulation. Patients reporting immediate or delayed onset of dizziness following the inciting trauma are included. None of the patient suffered from dizziness or vestibular symptoms prior to the injury.

All patients underwent a thorough neurotological examination, as well as audiological and vestibular evaluations. This included Siegel's pneumatic otoscopy in the clinic to elicit a Hennebert sign. Vestibular evaluation included a videonystagmography test battery (VNG). All patients underwent testing for subjective or objective Tullio and Hennebert signs. Cervical evoked myogenic potentials (cVEMPs) were added to the diagnostic test battery as these studies became available to our centers.

Audiological assessments included comprehensive audiometry (GSI Audiostar Audiometer, Eden Prairie MN), tympanometry (GSI Tympstar Tympanometer, Eden Prairie MN) in a sound-proof booth, videonystagmography (VNG) including positioning, and caloric irrigations (Micromedical VNG Visual Eyes 4 channel spectrum, Eden Prairie MN). Special testing was completed pre- and post-operatively on a per patient basis with some variability based on patient symptoms, falls risk, patient apprehension, and the examiner. A minority of patients were tested with cVEMPs pre-and post-operatively. There was a lower rate of follow-up (<50%) for vestibular assessment after surgery due to reported trepidation to repeat testing, loss to follow-up, and patient relocations.

Fistula assessments were utilized as the patient was seated, standing, or on a calibrated foam surface (NeuroCom Balance Manager Dynamic Platform Post-urography system) with vision denied and the use of video eye recording for most patients. Fistula assessments utilized tympanometry as well as ipsilateral and contralateral acoustic reflexes. Acoustic energy utilizing a standard probe tone of 226 Hz was introduced into an ear canal cavity by way of a loudspeaker and a microphone housed within a probe box. The ear canal cavity was hermetically sealed for testing. The pressure transducer, also housed within the probe box was utilized. Measures on tympanometry show maximum mobility of the tympanic membrane when pressure induced into the ear canal equals that in the middle ear (atmospheric pressure relative to middle ear pressure). If a patient reported dizziness during testing, this was a positive subjective Hennebert sign. Ipsilateral and contralateral acoustic reflexes were obtained at loudness intensities of 70–110 dB HL from 500 to 2,000 Hz with a hermetically sealed and pressurized ear from tympanometry recording. During presentation of the acoustic reflex stimuli, a subjective report of dizziness, or objective sway or eye-movements were reported as a positive Tullio phenomenon. Objective recording for nystagmus was also utilized in visually denied patients.

cVEMPs were performed using Bio-logic Navigator Pro Auditory Evoked Potential (AEP) system. The patient was placed supine in a MaxiSelect automatic chair. After skin preparation with 3M Red Dot™ Trace Prep and Nuprep skin gel, Natus jelly tab sensors were attached to Natus Alligator clips and placed in a one channel montage. One electrode was placed over the middle of the sternocleidomastoid (SCM) on the left and right. The ground electrode was placed at the forehead. Air-conducted rarefaction 500 Hz tone bursts were presented unilaterally via an ER 3A-insert earphone. The patient was given instructions to lift the head only and rotate it to the contralateral side producing tonic activation of the SCM muscle. Stimulus was presented at 95 dB nHL to the left ear first. cVEMP response thresholds were recorded from the ipsilateral SCM using a serial down by 10 dB procedure until a presentation stimulus of 65 dB was reached. The same process was then repeated on the right side.

All patients underwent high-resolution temporal bone CT scans without contrast looking specifically for temporal bone fractures, findings of site(s) of dehiscence in the bone of the otic capsule or congenital anomalies. Since intractable dizziness occurred following trauma a presumptive clinical diagnosis of traumatic perilymph fistula with perilymph leakage was made. Only those patients with positive Tullio and/or Hennebert tests were offered surgery. Retrocochlear pathology was ruled out using magnetic resonance imaging (MRI). One patient (Table 1, Patient 3) with bilateral superior semicircular dehiscence was included as she was completely asymptomatic prior to head trauma. cVEMPS were not performed in every case due to the lack of availability of the test early in our series.

**Table 1:** Demographics surgical findings and results.

**No**	**Age (yrs)**	**Symptom duration (MTS)**	**Possible etiology**	**Associated symptoms**	**Fistula sign**	**Surgery side**	**Findings**	**Results & notes**
1	31	26	Pain while snorkeling	Light headed, hyperacusis	AU AD>AS	AD	AD: membranous footplate, no perilymph leak	Complete Resolution, Occasional light headedness
2	55	12	Severe blow over occiput from falling door and frame	Headaches, “foggy feeling,” hyperacusis	AU	AU	AD: membranous in center of footplate, no perilymph leak. AS: membranous, no leak	Complete Resolution
3	69	21	Mild head trauma, possible osteoporosis, bilateral SSCD	Spinning sensation, hyperacusis	AD	AD	AD: stapes footplate intact but hypermobile, no perilymph leak	Complete Resolution
4	38	12	Motor vehicle accident	Hyperacusis	AU AD>AS	AD	AD: membranous footplate, no perilymph leak	Complete Resolution
5	36	30	Electric shock at work with fall at work striking head	Hyperacusis	AU AS>AD	AS	AS: membranous footplate, no perilymph leak	Complete Resolution
6	16	2	Weight lifting, concussions playing football	Migraine, light headed, hyperacusis	AS	AS	AS: footplate intact, perilymph fluid leak	Complete Resolution
7	50	26	Motor vehicle accident	Headaches, light headed, hyperacusis	AU AS>AD	AS	AS: footplate intact, perilymph fluid leak	Complete Resolution for 2 months. Now has migraine headaches and recurrent symptoms
8	41	15	Blast injury in Afghanistan, two concussions	Traumatic brain injury, post-concussive migraine	AU AD>AS	AD	AD: membranous defect in center of footplate, no perilymph leak	Complete Resolution, Occasional light headedness
9	39	11	Hit on right temple by engine block on conveyer belt	Cannot ride in an elevator, hyperacusis	AD	AD	AD: membranous footplate, no perilymph leak	Complete Resolution. Has problems tracking fast moving objects and has eye floaters
10	68	28	Possible osteoporosis	Spinning sensation, hyperacusis	AD	AD	AD: stapes footplate intact, perilymph leak	Complete Resolution
11	58	11	Multiple falls and concussion	Migraine, meniere, left BPPV, hyperacusis	AU AD>AS	AD	AD: stapes footplate intact, perilymph leak	Complete Resolution
12	56	21	Concussion, several falls, vehicle accident	Hyperacusis	AU AS>AD	AU	AS: membranous footplate, no perilymph leak, AD: membranous footplate, no perilymph leak	Complete Resolution. Twenty three months later patient involved in MVA. Resolved after revision surgery AS
13	16	32	Heat stroke and fall with occiput hitting concrete	Could not tolerate tuning fork test	AU AD>AS	AD	AD: membranous footplate, no perilymph leak	Complete Resolution
14	49	22	Barotrauma after flight, mild head trauma	Hearing fluctuates, sensation of passing out	AU	AU	AD: membranous footplate posterior half, no leak; AS membranous footplate no leak	Complete Resolution, able to fly to Belgium
15	60	25	Sudden left SNHL of uncertain etiology, possible mild trauma	Spinning sensation with pneumatic otoscopy	AS	AS	AS: two membranous defects in the footplate, no perilymph leakage	Complete Resolution for 7 months then recurred after a coughing fit. Managed conservatively
16	24	37	Head injury from falling out of second floor window	Suggestive of meniere disease, hyperacusis	AS	AS	AS: membranous footplate, with possible perilymph leak	Failed treatment. Two days following treatment lifted 46 lbs. Felt something pop in ears with recurrent symptoms. Lost to followup.
17	43	6	Head injury and unconsciousness from fall, car accident	Heavy metal exposure, history of BPPV	AD	AD	AD: footplate intact hypermobile footplate, no perilymph leak	Complete Resolution. One year post car accident, deployment of airbags, symptoms recurred but resolved completely
18	41	28	Multiple falls and minor head trauma	Migraine, scotomas, hyperacusis,	AD	AD	AD: membranous footplate, no perilymph leak	Complete Resolution. Cervical steal syndrome detected however
19	56	14	Head injury, found unconscious	History of meningitis and mastoiditis	AD	AD	AD: footplate intact, perilymph leak present	Complete Resolution
20	46	62	Severe car accident with loss of consciousness	Hyperacusis, tired, cannot fly	AS	AS	AS: intact footplate with possible perilymph leak	Complete Resolution. Able to fly
21	54	23	Severe car accident with whiplash and loss of consciousness	Spinning, hyperacusis, neck surgery, headaches	AU AS>AD	AS	AS: perilymph fluid contained in sac, possible perilymph leak, but footplate intact and hypermobile	Partial Resolution. Did not keep follow-up
22	27	18	Car accident with whiplash	Hyperacusis with Tullio and Hennebert	AD	AD	AD: irregular membranous deficiency in footplate, hypermobile but no perilymph leak	Complete Resolution
23	54	28	Car accident with whiplash and concussion	BPPV treated, fogginess, hyperacusis	AD	AD	AD: membranous dehiscence in bony footplate, no perilymph leak	Complete Resolution. One week post-op had minimal symptoms which resolved
24	34	30	Car accident with whiplash and multiple fractures and head trauma	BPPV treated, fogginess, hyperacusis	AU	AU	AD: Crack in stapes footplate, no perilymph leak, AS: crack in stapes footplate but perilymph leak at round window	Partial resolution. Patient feels much better but not quite normal. Lost to follow-up
25	24	43	Struck by car and fall from jet ski	BPPV treated, hyperacusis, dysautonomia	AD	AD	AD: crack in footplate, no perilymph leak	Failed treatment. Stayed symptomatic, postural hypotension
26	50	21	Blow to head	Hearing cell phone causes nystagmus	AD	AD	AD: membranous defect in footplate, no perilymph leak	Complete resolution after revision surgery. (Patient had coughing fit 2 weeks after surgery with recurrent symptoms)
27	22	6	Blow to ear with baseball glove	Falls to right with noise. 30% difference in VEMP	AD	AD	AD: ? crack in footplate, no perilymph leak, hypermobile stapes footplate	Complete Resolution
28	62	27	Blow wit football on right side	“Fun house” bending forward results in fall, staggering	AS	As	AS: membranous footplate, no perilymph leak	Complete Resolution. Immediate resolution 24 h post-surgery. VEMP normalized prominent OTR

*?, questionable finding*.

The gray-scale inversion or “invert” function on the PACS (picture archiving and communication system) which uses the universal DICOM® (Digital Imaging and Communication in Medicine) was used to improve visualization of the stapes footplate on high resolution temporal bone CT scans. Patients who had persistent signs and symptoms suggestive of TWS were offered exploratory surgery. In patients suspected of bilateral pathology the more symptomatic side was operated upon first. If symptoms were controlled or resolved the second side was not operated upon. Exploratory surgery was offered only after a prolonged period of failed conservative management and after a detailed informed consent was obtained.

Middle ear exploration was performed under general anesthesia using both the Carl Zeiss OPMI Pentero 800 microscope (Carl Zeiss Meditec Inc. Dublin, CA) and 4 mm sinus endoscopes (Karl Storz Endoscopy-America, Inc. CA). Still photographs and video recording system using the integrated microscope video camera or a Storz 3-CCD camera (Karl Storz Endoscopy-America, Inc. CA) were used for photo and video documentation. A transcanal exploratory tympanotomy approach was used and the posterior superior bony canal overhang was removed with a curette in order to visualize the oval and round window niches. Mucosal adhesion bands obscuring visualization of the footplate and round window membrane were lysed. Intraoperatively we looked for the presence or absence of perilymph leakage. If none was present, 3 sequential Valsalva maneuvers lasting 5 s were performed by the anesthesiologist 1 min apart. After observing membranous stapes footplates in the first few cases, we began to actively look for their presence in subsequent patients. If present paradoxical movement of the membrane was elicited by gently balloting the posterior crus of the stapes using a Rosen pick or Derlacki mobilizer (Video 1). Patients were classified as having a perilymph leak if fluid could be seen actively pooling in the round or oval window niches. A questionable leak was diagnosed if a small amount of fluid was present which did not refill after suctioning despite multiple Valsalva maneuvers. If neither were present a perilymph leak was ruled out. In symptomatic patients, if a membranous stapes footplate or a perilymph leak was not observed, the diagnosis of a hypermobile stapes footplate was inferred.

In all cases the mucosa around the oval and round window niches were denuded. Tiny fat grafts harvested from the cranial aspect of the ear lobule, were packed under the arch of the stapes, and anterior and posterior to the stapes crurae, and also in the round window niche. Gelfoam® (Pharmacia and Upjohn, Kalamazoo, MI) soaked in saline was placed in the middle ear to stabilize the grafts and the tympanomeatal flap was replaced. A small amount of Gelfoam® (Pharmacia and Upjohn, Kalamazoo, MI) soaked in saline was then placed over the incision site.

Post-operatively patients were placed on bed rest and stool softeners for 2 weeks. Patients were cautioned against vigorous nose blowing, straining, and lifting weight heavier than 10 lbs. Follow-up examination was performed in 1 week. At 6–8 weeks the patient's impressions were recorded and a clinical exam included pneumatic otoscopy and an audiometric evaluation was performed. Post-operative testing in the vestibular laboratory was ordered at 2 months.

### Illustrative Case 1

A 36-year old male factory worker (Table 1, Patient 5) was seen on 15 January 2013 with a 2.5 year history of dizziness and imbalance. The dizziness began a few weeks following a severe electrical shock at work, which caused a fall and he struck his occiput on the concrete floor. This was accompanied by a brief period of unconsciousness, however intracranial hemorrhage was ruled out. The patient was initially diagnosed as suffering from benign paroxysmal positional vertigo (BPPV) and underwent several Epley (4) canalith repositioning maneuvers without relief. Medical therapy for migraine variant dizziness (vestibular migraine) was also unsuccessful. He had not worked for over 2 years. He reported a constant dizzy sensation during all waking hours. Dizziness was associated with headaches, and irritability to both light and sound. Looking up or down and rotation of his head exacerbated his symptoms. Bending down in particular, performing a Valsalva maneuver, and something as trivial as traveling in an elevator worsened his symptoms. He reported the perception of a tilted horizon. Changes in atmospheric pressure such as an incoming storm caused ear fullness and increasing dizziness. He was unable to ride a bicycle. He also reported having to concentrate to avoid falling and feeling exhausted and irritable at the end of the day. As a result he reported feeling extremely depressed and confessed to suicidal ideation.

**Figure 1:**
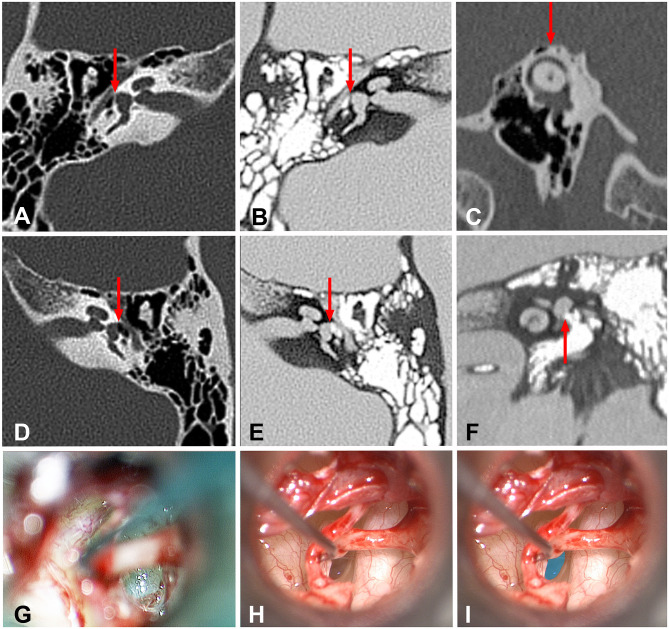
High resolution temporal bone CT images of the normal and abnormal stapes footplate as well as intraoperative images of the hypermobile stapes footplate. **(A)** Traditional axial image of the right temporal bone with bone windows shows the cochlea, internal auditory canal, vestibule, middle ear. Red arrow shows the normal stapes footplate (white line) providing the interface between the vestibule and middle ear. **(B)** Same image as seen in this figure **(A)** using the “invert” function. Note the ease in comparing the air in the middle ear (white) to the fluid-filled vestibule (gray). Red arrow shows the position of the normal stapes footplate (black line) providing the interface between the vestibule and middle ear. **(C)** Traditional Poschl image of the left temporal bone with bone windows shows that in this case of left third window syndrome, there is no superior semicircular canal dehiscence (red arrow). **(D)** Traditional axial image of the left temporal bone with bone windows shows the cochlea, internal auditory canal, vestibule, middle ear. Red arrow shows the position of the membranous stapes footplate providing the interface between the vestibule and middle ear. Note the absence of the white line seen in this figure **(A)**. **(E)** Same image as seen in this figure **(D)** using the “invert” function. Note the ease in comparing the air in the middle ear (white) to the fluid-filled vestibule (gray). Red arrow shows the position of the membranous stapes footplate providing the interface between the vestibule and middle ear. Note the absence of the black line representing the normal stapes footplate seen in this figure **(B)**. **(F)** Inverted coronal image of the left temporal bone with bone windows shows the cochlea, vestibule and middle ear. Red arrow shows the position of the membranous stapes footplate providing the interface between the vestibule and middle ear. Note the absence of the black line representing the normal stapes footplate. **(G)** Intraoperative appearance of a normal stapes footplate. **(H)** Intraoperative appearance of a membranous stapes footplate. Note the translucent appearance of the entire stapes footplate. The bone-covered tympanic segment of the facial nerve can be seen to the right of the stapes footplate. **(I)** The stapes footplate is shaded in blue to illustrate the position of the membranous stapes footplate image seen in this figure **(H)**.

The patient held his head tilted to the left. Pneumatic otoscopy in the left ear elicited nystagmus associated with nausea. An audiogram demonstrated slightly asymmetric 4 kHz “noise notches” in both ears, but was more pronounced on the left side. Tullio and Hennebert tests were performed in the vestibular lab and found to be positive. The patient reported the sensation of being pushed to the right. Clinically, the possibility of a TWS or a perilymph fistula was suspected. A high resolution temporal bone CT scan was interpreted by the neuroradiologist as being normal. However, one of the authors (AKG) found that a bony defect was observed in the left stapes footplate when compared with the right. This became more apparent when the “invert” function was used on the PACS system (Figure 1). On 23 May 2013, he underwent middle ear exploration and a floppy membrane appeared to have replaced the bony stapes footplate. There was no evidence of perilymph leakage. Fat grafts from the lobule were used to reinforce both the round and oval windows as previously described. At follow-up examination in 8 weeks the patient reported that his symptoms had completely resolved (Video 2). Hearing fluctuation and noise intolerance resolved completely and he was able to ride a bicycle. Due to a fear of symptom recurrence the patient refused post-operative objective testing. He requested to be released back to work, and was symptom-free for 12 months when he was lost to follow-up.

### Illustrative Case 2

A 27-year-old female audiologist (Table 1, Patient 22) suffered a whiplash injury after being involved in a motor vehicle collision in February 2014. In August 2014 she began complaining of dizziness which increased progressively. Although her symptoms were not consistent with benign paroxysmal positional vertigo, she underwent several canalith repositioning maneuvers at another institution without relief. In December 2014, she felt extremely nauseated on an airplane flight. She was first seen in our office in June 2015. In addition to intermittent dizziness, she complained of right aural pressure and pain, and an intolerance to loud sounds. Additionally, she also reported cognitive difficulty, and an inability to concentrate on her work. Changing altitude while driving over hilly terrain was particularly disorienting. There was no complaint of a hearing loss or tinnitus. A fistula test using pneumatic otoscopy resulted in nystagmus. The Tullio test was positive and a post-traumatic perilymph fistula was suspected. A high resolution CT scan of the temporal bones was performed and reported as being normal. She consented to right middle ear exploration and surgery was performed on 20 August 2015. Perilymph leakage was not encountered; however, a bony defect in the stapes footplate covered by a thin membrane was observed and patched using fat grafts. At follow-up examination in 8 weeks the patient reported that her vertiginous symptoms had completely resolved, and conductive hearing loss following surgery returned back to baseline (Video 3). She also reported a resolution of cognitive issues and was able to test high powered hearing aids as part of her professional duties without suffering dizziness. The patient has been symptom free for 28 months. This was confirmed in the vestibular laboratory.

### Illustrative Case 3

**Figure 2:**
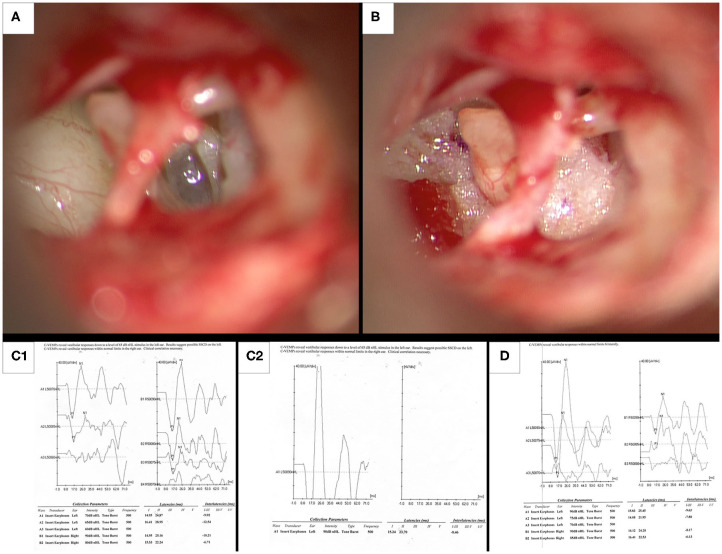
Intraoperative photographs and preoperative and post-operative cVEMP responses in illustrative case 3. **(A)** Intraoperative appearance of a membranous translucent left stapes footplate. **(B)** Fat grafts obtained from the lobule are placed under the arch of the stapes and anterior and posterior to the crurae. **(C1,C2)** Preoperative cVEMPs on the left ear with increased amplitude and decreased threshold down to 65 dB nHL, along with normal cVEMPs in the right ear. **(D)** After auditory thresholds had normalized at 8 weeks following surgery, post-operative cVEMPs shows normalization of thresholds and amplitude on the left side. This suggests that surgery resulted in objective improvement in cVEMPs.

A 62-year-old female patient (Table 1, Patient 28) presented to our office on 6 June 2019 with a history of dizziness. She reported being struck in the head by a football in September 2017. There was no history of loss of consciousness, but she was seen in the emergency room and was diagnosed with concussion. Soon after the event, she began developing progressively increasing dizziness. She described a sensation of being pushed and had a feeling that she would fall when exposed to something as trivial as a wind gust. During all waking hours she described a feeling of being in a “funhouse,” and was extremely unstable on her feet. She was unable to go for rides on her motorcycle because the low-pitched exhaust sounds bothered her. Rolling over in bed occasionally made her dizzy. Dizziness was most pronounced on exposure to loud sounds, and changes in altitude such as riding in an elevator were especially bothersome. While walking she needed to hold on to the wall or her spouse for support. Her symptoms were most pronounced when bending down. She reported an inability to prevent a fall when bending to pick up an object from the floor. Multiple sessions under the care of a physical therapist did not relieve her symptoms. Ear, nose and throat examination was normal, however the patient reported extreme nausea on left-sided pneumatic otoscopy, and a questionable left beating short-lived nystagmus was observed. Bedside vestibular testing and VNG were found to be normal on 9 August 2019. The Hennebert test was equivocal, but with the Tullio test the patient demonstrated pulsion to the right. cVEMP was normal on the right but was abnormal on the left side with increased amplitude and decreased threshold down to 65 dB nHL (Figures 2C1,C2). The gray-scale inversion technique on high resolution temporal bone CT scan raised the possibility of a left membranous stapes footplate. The patient consented to surgery and on 5 December 2019 underwent fat graft placement over the oval and round windows (Figures 2A,B). While her ear felt plugged following surgery her symptoms of disequilibrium resolved almost immediately. On 14 January 2020 pulsion was not elicited on Tullio testing. Auditory thresholds recovered completely to preoperative levels, and post-operative cVEMP testing demonstrated normal amplitude and threshold on the left side (Figure 2D). The patient's and her husband's impressions can be seen and heard in Videos 4, 5. The patient reports that she is almost completely back to normal.

## Results

All 28 patients (32 ears) had some level of noise intolerance or hyperacusis associated with varying degrees of dizziness, imbalance, or vertigo. There were 7 males (25.0%) and 21 females (75.0%), whose ages ranged between 16 and 69 years. Our findings are summarized in Table 1. All patients had varying degrees of head trauma; In 18 of 28 patients (64%) the trauma could be considered severe with evidence of concussion, or unconsciousness. Audiometric data was variable with no consistent pattern. Most patients developed symptoms several weeks to months following the trauma. Three patients developed immediate symptoms. One patient with prior history of concussions exhibited symptoms while power-lifting. He was found to have a perilymph leak but the stapes footplate was devoid of defects (Video 6). Two others patients with immediate onset dizziness (one was struck with a baseball glove on the ear, and another who was hit in the head by a football) did not demonstrate perilymph leakage. One patient in the series also reported a blast injury while in the military. Only one patient (Table 1, Patient 3) demonstrated evidence of bilateral superior semicircular canal dehiscence syndrome in addition to a defect in the stapes footplate. The duration of dizziness varied from 2 months in a patient who developed a perilymph fistula while power lifting to 62 months in a patient who was the victim of a severe car accident. The mean duration of symptoms was 22.8 months.

On high resolution temporal bone CT scan none of the patients had identifiable congenital temporal bone anomalies. It is worth mentioning that when the “invert” function was used to visualize the stapes footplate, most patients were observed to have bony defects of variable size on one or both ears. Some of these defects appeared to be subtle gaps or simply cracks without perilymph egress at the time of surgery.

Positive Hennebert or Tullio signs were present in all patients; 12 patients bilaterally (AU), while 11 patients had right-sided (AD), and 5 had left-sided (AS) involvement. Sixteen patients underwent right-sided explorations, 8 had surgery on the left, while 4 had sequential surgery on both ears. Two patients in this series (Patient 12 and Patient 14), required revision surgery as symptoms returned; one following a vehicular accident and the other following a severe bout of coughing. In both, the fat graft had displaced, and their symptoms resolved after revision surgery with new fat grafts being placed.

At the time of surgery, 21 of the 32 (65.6%) ears had what appeared to be bony defects in the stapes footplate which were covered over by a translucent membrane. Some of these defects could be very small and in 4 ears (12.5%) cracks in the footplate without evidence of perilymph leakage were noted. Only 7 of the 32 (21.9%) ears showed evidence of true perilymph leakage (5 involved the oval window, 1 the round window and 1 involved both round and oval windows). The remaining 4 ears (12.5%) had neither leakage of perilymph nor was a membrane present despite being symptomatic. These were determined to have hypermobile stapes footplates. They all exhibited positive Hennebert signs and Tullio phenomena but the stapes footplates were devoid of observable cracks, defects, or membranes. Four ears (12.5%) demonstrated questionable perilymph leaks, and the majority, i.e., 21 ears (65.6%) did not demonstrate any evidence of perilymph leakage. Five of the 7 ears (70.4%) with evidence of perilymph leakage had intact stapes footplates. In contrast only 1 of the 21 ears with membranous footplates showed evidence of perilymph leakage (4.8%).

One patient (Table 1, Patient 3) demonstrated a right membranous footplate in addition to bilateral SSCD. She had Tullio and Hennebert signs which were positive on the right side and her symptoms resolved after patching the right oval and round window niches. Therefore, we did not operate on the left ear and did not need to address the radiographic finding of SSCD.

cVEMP testing was performed in 17 ears. Of the 17 symptomatic ears tested, 13 (76%) had subnormal thresholds on cVEMPs, while 4 of the 17 (24%) showed normal thresholds preoperatively. Of the 13 patients with abnormal cVEMPs, 9 (69%) of them had normal threshold results post-operatively. We were unable to obtain post-operative cVEMPs on 4 ears (31%). Post-operatively pneumatic otoscopy was performed on all patients in the clinic; however, several patients did not consent to undergo the Hennebert or Tullio testing in the laboratory because of their anxiety that their symptoms would return. We were able to obtain vestibular post-operative testing in 14 of the 28 patients. Clinically 24 of the 28 patients (85.7%) showed complete amelioration of symptoms and no cases of hearing deterioration occurred in this series. Four of the 33 ears (12.1%) failed to show improvement following surgery.

## Discussion

It is widely accepted that BPPV is the most common cause of vertigo after head injury (1, 5). However, we observed that there is a cohort of patients who appear to resist conventional canalith repositioning maneuvers and go on to develop symptoms of persistent incapacitating recalcitrant dizziness. It often becomes de rigueur to label these patients as having post-concussive syndrome (PCS), or chronic traumatic encephalopathy (CTE). It was only after we started obtaining Hennebert and Tullio tests that we began to realize that there could be another etiology for symptoms in addition to BPPV, and post-traumatic vestibular migraines. Hoffer et al. (6) found that after mild head trauma individuals suffered in descending order of frequency from post-traumatic vestibular migraines, post-traumatic positional vertigo, and 19% were classified as suffering from post-traumatic spatial disorientation. This last group was distinguished from the others by a lack of migraine headaches, a constant feeling of unsteadiness, and abnormalities on static posture testing. They also speculated that individuals who did not recover within 1 year after the injury may have a different pathology and require different treatment modalities. These findings are remarkably similar to patients in our series. However, Tullio and Hennebert tests and the possibility of TWS were not considered as part of their study. The mean duration from the onset of symptoms to getting treated in our series was almost 2 years. The outlier in our series who sought treatment within 2 months was a power weight-lifter who reported hearing a pop in the ear associated with an immediate onset of disequilibrium. The longest duration of symptoms was 62 months in a patient who suffered a severe car accident with life threatening injuries and had unsuccessfully sought care at several institutions. She was diagnosed as having TBI and was forced to resign from employment. Unfortunately she had never been tested for a TWS. Fat grafting of the round and oval windows ameliorated her symptoms.

Tullio and Hennebert tests are known to be positive in several otologic conditions. These include TWS (e.g., superior semicircular canal dehiscence or cochlea-facial nerve dehiscence), perilymph fistula, Menière disease, post-fenestration surgery, vestibulofibrosis, (7) vestibular atelectasis, (8, 9) and also otosyphilis. The diagnosis and management of spontaneous perilymph fistulas is extremely controversial (10). Several prominent surgeons have questioned their existence and surgical repair of the oval and round windows for the management of spontaneous perilymph fistula leaks remains controversial (11, 12). This is not the case for acquired or post-traumatic perilymph fistulas.

It is well-known that post-traumatic perilymph fistulas of the round and oval windows can occur after minor trauma without skull fractures (13, 14) Small defects can occur with dizziness and a positive Hennebert sign despite hearing being normal (15). The symptoms as we found in our series can be quite variable (16, 17). Victor Goodhill (18) first proposed the theory of implosive and explosive forces causing acquired perilymph fistulas. We believe that most subjects in our series were the result of implosive forces. The exceptions were the power lifter who developed symptoms on straining and the patient who required revision surgery due to coughing.

House (19) observed that surgical exploration was needed to detect perilymph leakage in post-stapedectomy cases. To this day preoperative detection of the site of leakage remains problematic. Tullio test and Hennebert sign can provide a clue that a fistula or TWS may be present but they do not point to a precise site of leakage. Given the history of prior cranial trauma we performed all surgical explorations presuming the diagnosis of perilymph leakage from the inner ear. All patients were operated upon after failed conservative management. At the time of surgery perilymph leakage could be detected in only a minority of cases (21.9%). Instead a membranous stapes or a hypermobile stapes footplate without perilymph leakage was observed in the majority of cases (78.1%). This was unexpected.

A pliable, compliant membranous footplate also appears to protect against perilymph leakage, or may perhaps represent spontaneous healing of an injured footplate. When the footplate was membranous only 4.8% of ears showed demonstrable perilymph egress. Conversely, of the ears with intact footplates 70.4% showed the presence of perilymph leakage. Patients with hypermobile stapes footplates represented a not insignificant minority (12.5%) of explored middle ears. It is noteworthy that they exhibited all clinical characteristics of membranous stapes footplates preoperatively. A mechanical or electronic method to objectively document hypermobility is being explored.

Isolated congenital dehiscence of the stapes footplate has been described in the literature but is exceedingly rare, and usually seen in Mondini deformity (20, 21). We therefore believe that the dehiscences seen in all of our patients were acquired following head trauma. Therefore, our principle hypotheses are: (1) rapid acceleration and deceleration may result in a temporary subluxation of the stapes footplate with disruption of the tiny capillaries supplying the region; and (2) gradual avascular necrosis results in variable sized defects that develop over time. This would also explain the delayed presentation of symptoms in some of our patients. An alternative hypothesis is that trauma results in a fracture which goes on to heal spontaneously. Schucknecht (22) has alluded to similar pathology in human temporal bones and experimental animals, and recommended the early use of connective tissue to patch the defect. He made a similar observation after radiation therapy with an accompanying atrophy of the annular ligament (22).

The work of Minor et al. (23) and Minor (24) in 1998, spawned a great deal of interest in dizziness associated with defects in other areas of the otic capsule. Wackym et al. (3, 25) coined the terms otic capsule dehiscence and third window syndrome (TWS) to describe the spectrum of signs and symptoms observed in these patients. This all-encompassing term correctly alludes to the fact that several other defects in the otic capsule can result in symptoms and a phenotype of the spectrum seen in SSCD patients. The stapes footplate is developmentally part of the otic capsule bone and congenital or acquired dehiscence in the stapes footplate can conceivably represent yet another manifestation of TWS.

The mechanisms for dizziness in membranous or hypermobile stapes footplates has not been well-elucidated. In 1883, Gellé (26) was able to associate dizziness with mobility of the stapes and pathology of the oval and round windows. In 1905, Hennebert (27) demonstrated oculo-vestibular disturbance by changing pressure in otherwise normal appearing ears. A fistulous communication between the perilymph and the middle ear was suspected, but in the absence of magnification could not be demonstrated (28). Bárány (29) therefore concluded that Hennebert's sign was the result of increased stapes mobility.

Dieterich et al. (30) studied a 35-year-old professional horn player who developed an excitatory ocular tilt reaction (OTR), and balance disturbance by tones of 480 +/– 20 Hz at 95 dB. It was manifested as an ipsilateral head tilt, skew deviation of the eyes and ocular torsion, and was characterized as an otolith Tullio phenomenon. At the time of surgery a medially subluxed stapes footplate with hypertrophic stapedius muscle was discovered. However, they did not report the presence of a membranous stapes footplate. The patient's symptoms resolved completely when compressed silastic foam was inserted between the anterior and posterior crurae of the stapes. We utilized the same principle but used autologous lobule fat instead. The presence of fatty tissue perhaps changes impedance and may prevent large medial-lateral displacement of an acquired membranous or hypermobile stapes footplate. In order to make sure that imperceptible perilymph leaks were not missed packing was also performed anterior and posterior to the crurae of the stapes and in the round window. Twenty-four of 28 patients (85.7%) showed complete resolution of symptoms following surgery. Four of the 28 patients failed surgical treatment. These patients may represent an opportunity for future refinement in diagnosis and treatment.

Backous et al. (31) described the relationship of the stapes footplate to the utricle and saccule in 130 temporal bones. However, in none of the specimens was the footplate in contact with either structure. Membranous connections between the utricle and footplate were seen in 34 bones, and pulsion or traction may help explain the otolith Tullio phenomenon. In our series a floppy membrane or hypermobile stapes footplate touching the utricle directly remains a possibility. Alternatively, post-traumatic adhesions may form between the stapes footplate and membranous labyrinth causing symptoms.

Ehmer et al. (32) described the “bulging oval window” sign on CT and MRI. This represents an out-pouching of a fluid filled sac in the region of a stapes footplate defect. We did not specifically look for this sign in our series, but serendipitously found that by using the gray-scale “invert” function, small defects in the stapes footplate could be more readily visualized. Images on CT scan are technically negatives, with air appearing black and bone appearing white. Gray-scale inversion renders a positive image. The physiology literature describes that the human visual system demonstrates optimal contrast perception when a dark object is visualized against a bright background (33). Formal studies utilizing radiologists and otolaryngologists blinded to the diagnosis are necessary to evaluate the sensitivity and specificity of the gray-scale “invert” function as it pertains to this pathology of the stapes footplate. We are further investigating the utility of this technique to visualize other temporal bone pathologies, which is the subject of another paper. It is our current recommendation that CT scans with the “invert” function, must be utilized in the context of the clinical picture, and should not be used in isolation to make the diagnosis or to recommend surgery.

An inherent limitation of this study is its retrospective nature. An additional limitation is that preoperative vestibular evoked myogenic potentials (cVEMPs) and ocular vestibular evoked myogenic potentials (oVEMPs) were not routinely performed. This was due to the fact that given the clinical history of trauma we were looking for perilymph leaks rather than TWS. Additionally, at the start of our work we did not have the necessary equipment to perform these tests. An additional barrier to cVEMP testing may have been lack of reimbursement for the test. Out of pocket costs may have precluded patient participation in post-operative testing. A multi-institutional prospective study to better characterize ocular movements, and hopefully differentiate between other defects producing symptoms of TWS and membranous and hypermobile stapes footplates is warranted. In illustrative Case 3, (Figure 2) we were able to demonstrate a normalization of cVEMP following surgery. Consequently, a systematic use of cVEMPs and oVEMPs is a subject for further research.

While all patients underwent post-operative pneumatic otoscopy in the office, we were able to convince only 50% of our patients to obtain post-operative objective testing. Most patients appeared too nervous to get retested, while three patients had relocated from the area. The use of Tullio and Hennebert tests in post-traumatic dizziness must be encouraged.

## Conclusion

It is hoped that this article draws attention to an underappreciated clinical entity of post-traumatic membranous and hypermobile stapes footplates that result in symptoms of TWS, including perilymph fistulas. Its pathophysiology and management is discussed. It can cause persistent and often intractable dizziness following head trauma which is often mistaken for traumatic brain injury or post-concussive syndrome. In our experience the presence of true perilymph leak is a relatively uncommon event. Tullio and Hennebert tests help to clue in on the diagnosis and should be performed in every case of persistent dizziness after head trauma. Imaging modalities such as high resolution temporal bone CT scans combined with gray-scale inversion should not be used in isolation but rather used in the context of the clinical history, physical findings and objective audiological and vestibular tests. Fat grafting of the oval and round windows is a low risk procedure with a high probability of curing the condition.

## Data Availability Statement

The raw data supporting the conclusions of this article will be made available by the authors, without undue reservation.

## Ethics Statement

The studies involving human participants were reviewed and approved by Heuser Hearing Institute Institutional Review Board. Written informed consent for participation was not required for this study in accordance with the national legislation and the institutional requirements. Written informed consent was obtained from the individual(s) for the publication of any potentially identifiable images or data included in this article.

## Author Contributions

AG: discovered the condition and performed surgeries and wrote the paper. IE: performed preoperative and post-operative audiology testing and helped analyze data at Heuser Hearing Institute. VB: performed pre and post-operative cVEMP testing and helped analyze data. CR: performed per and post-operative audiology testing and helped edit the paper. All authors contributed to the article and approved the submitted version.

## Conflict of Interest

The authors declare that the research was conducted in the absence of any commercial or financial relationships that could be construed as a potential conflict of interest.
